# Psychological effects of horizontal price display: how left-right location shapes reference price and perceived quality

**DOI:** 10.3389/fpsyg.2025.1497372

**Published:** 2025-02-19

**Authors:** Eun Young Park, Jung Min Jang

**Affiliations:** ^1^College of General Education, Kookmin University, Seoul, Republic of Korea; ^2^Brunel Business School, Brunel University of London, London, United Kingdom

**Keywords:** price promotion, anchoring and adjustment theory, reference price, location effect, product quality

## Abstract

This study examines the impact of display locations of regular and sale prices on reference price estimation, drawing on anchoring and adjustment theory and the left-to-right directionality in reading habits. It focuses on how the spatial positioning of the regular price relative to the discount price affects perceived quality and subsequently shapes reference price judgments. Conducted across both offline and online settings using varied product stimuli, three laboratory studies using comparative price advertisements that presented both prices on the same page consistently demonstrate that placing the regular price to the left of the discount price results in higher reference price estimations. The findings also confirmed that perceived product quality mediates the proposed effect. This research offers new insights into how the spatial placement of pricing information significantly affects consumer perception and decision-making, contributing to consumer psychology.

## 1 Introduction

In today’s competitive market environment, instore promotions and price discount tactics are crucial strategies for both online and offline marketing practitioners to attract customers, boost sales, and manage inventory effectively (Ahmetoglu et al., 2014; Competera, 2023). These tactics often involve reducing the initial cost of products or services to a more attractive sale price (Mastercard Services, 2024). Marketing practitioners utilize these strategies not just to draw in customers but also to communicate value effectively through strategic price comparisons—regular, original, or manufacturer’s suggested prices are often compared with sale prices to underscore the deal being offered. This approach leverages consumer psychology, enhancing the appeal of purchases by framing the sale price against a higher original price, thereby suggesting substantial savings (Della Bitta et al., 1981; Kalyanaram and Winer, 2022).

To optimize these communications, marketing practitioners must decide not only the extent of the price reduction but also the most effective methods for conveying these reductions to consumers (Della Bitta et al., 1981). The current study focuses on the latter—how marketing practitioners present the comparative price offer. This aspect is more straightforward for them to control as it involves fewer constraints compared to determining the discount depth, which directly affects net profits.

Delving deeper into the issue, we examine the nature of comparative price advertisements, which typically contrast regular prices against suggested sale prices (Chandrashekaran, 2004; Della Bitta et al., 1981; Grewal et al., 1998). This exploration underscores a critical aspect of our research: the horizontal positioning of price information. We focus on this dimension because it represents one of the most common methods of presenting comparative price offers in marketing communication environments. Specifically, our research centers on how the left-versus-right display of regular and discount prices can influence consumers’ psychological responses—such as their quality perceptions and reference price judgments—beyond the immediate appeal of a “good deal”.

Recent studies show that consumers’ reactions are significantly affected by the placement and order of price information (Bagchi and Davis, 2012; Biswas et al., 2013; DelVecchio et al., 2009; Jang and Park, 2020). For example, the attractiveness of a deal and purchase intentions are enhanced when the regular price is positioned to the left, rather than the right, of the discount price. This enhancement is due to easier computation and a congruence effect linked to temporal associations in pricing and horizontal positioning (Biswas et al., 2013; Jang and Park, 2020). However, while most research has concentrated on the deal attractiveness and purchase intentions as outcomes (Bagchi and Davis, 2012; Biswas et al., 2013; DelVecchio et al., 2009; Jang and Park, 2020), our study addresses a different concern: the potential risks of indiscriminate price discounts. Misused price discounts can cause consumers to lower their reference prices and devalue a brand; the product is judged and perceived as expensive; posing significant risks to store image and repurchase intentions (i.e., Graciola et al., 2018). Moreover, offering benefits without a price discount, such as a freebie for a period followed by selling at the usual price, typically does not devalue the product as much as a price discount because it helps to prevent the formation of a lower reference price (Ahmetoglu et al., 2014; Palmeira and Srivastava, 2013).

Recognizing the potential risks associated with price promotions, our study investigates whether the horizontal spatial format of comparative price presentations can mitigate their detrimental effects on consumer perception. Grounded in anchoring and adjustment theory (Tversky and Kahneman, 1974), this research explores how displaying the regular price on the left (versus right) of the discount price may enhance perceived quality and foster more favorable (i.e., higher) reference price judgments. Specifically, our study first aims to examine the influence of left-versus-right placement of regular and discount prices on consumers’ reference price judgments and, second, to investigate whether perceived product quality mediates the relationship between price display location and reference price judgments.

By introducing reference price judgment as a novel key dependent variable, along with perceived product quality, this study broadens the research scope in comparative price promotions. Our approach reveals how strategic manipulation of the horizontal price presentation can modify the anchoring process and ultimately influence consumers’ long-term valuation of a product as well as their price perception. This work advances the consumer psychology literature by deepening our understanding of how horizontal price displays shape perceptions, attitudes, and behaviors. We also offer practical guidance for marketing practitioners on designing more effective price promotions that optimize both perceived value and brand equity.

## 2 Theoretical background

### 2.1 Anchoring and adjustment theory

Anchoring and Adjustment Theory explains how individuals, when making decisions under uncertainty, tend to rely on initial pieces of information, referred to as anchors, and then make subsequent adjustments, which can be insufficient, based on this anchoring effect (Einhorn and Hogarth, 1986; Tversky and Kahneman, 1974). This phenomenon has been consistently observed, with numerical anchors profoundly influencing subsequent judgments (Barbera et al., 2018; Epley and Gilovich, 2006, 2010; Oppenheimer et al., 2008; Tversky and Kahneman, 1974). For instance, Tversky and Kahneman (1974) demonstrated that under time constraints, participants’ estimates for calculations like factorial 8! varied dramatically depending on the sequence of number presentation, whether ascending (1 to 8) or descending (8 to 1). Specifically, descending sequences consistently yielded higher estimates because the initial piece of information is ‘8’, which is larger than ‘1’ in ascending sequences. Expanding on traditional contexts, recent studies have explored how environmental cues, such as visual indicators of temperature (31, 32, 39, 81, 83, 89), can also function as psychological anchors (Barbera et al., 2018). Findings suggest that visual cues indicating outside temperature can anchor consumer valuations of services, such as accommodations, where perceptions of value increased with higher temperatures and decreased with lower ones. This extension of anchoring effects into environmental contexts underscores the theory’s broad applicability and its relevance in understanding how subtle cues influence consumer perceptions and decisions.

The theory suggests that initial information typically acts as an anchor, shaping initial judgments from which individuals make subsequent adjustments. These adjustments often remain insufficient, even when relevant additional data is present. This anchoring effect is observable in diverse contexts: from package (multi-item) price presentations (Bagchi and Davis, 2012), and evaluations of service products (Barbera et al., 2018) to price negotiations between buyers and sellers (Galinsky and Mussweiler, 2001), the order of menu presentations, whether descending or ascending (Suk et al., 2012), and the influence of physical anchors (LeBoeuf and Shafir, 2006). Such examples underline the significant role of anchors in decision-making processes, a fundamental aspect of anchoring and adjustment theory (Epley and Gilovich, 2010).

In the realm of comparative price advertisements, regular and discount prices are presented as two distinct pieces of numerical information, either of which might act as an anchor. This study explores how the positioning of these prices—whether the regular price or the discount price is presented first—affects their potential as anchors. These anchors significantly influence the subsequent decision such as formation of internal reference prices, where the initial price seen could set a cognitive baseline that impacts all subsequent price evaluations.

The main question of this research is determining which price, regular or discount, serves as the more dominant anchor and how this influences consumer perceptions and decision-making when identical price information is offered. We hypothesize that the horizontal display order of these prices may alter their impact as anchors, affecting how consumers internally assess value. This is explored further in subsequent sections where we investigate how different display locations of regular and discount prices shape internal reference prices.

### 2.2 Directionality of attention and horizontal price locations

Research has consistently shown that the directionality of attention is influenced by habitual patterns such as reading from left to right, which affect how people process sequences and spatial layouts (Chatterjee et al., 1999; Christman and Pinger, 1997; Santiago et al., 2007; Santiago et al., 2008). When people visualize or engage with sequences of events or actions, they typically imagine them moving leftward to rightward. This left-to-right bias influences how individuals perceive and process visual information, including how they view sequences outlined in images, text, and even pricing information. Moreover, the abundance of cultural symbols that utilize left-to-right sequencing, such as written text and graphs, reinforces this bias in visual attention (Santiago et al., 2007; Mendonça et al., 2022). This directional bias commonly leads to superior attentional performance for objects or information that start on the left and move towards the right (Maass and Russo, 2003; Ouellet et al., 2010; Spalek and Hammad, 2005), demonstrating that habitual eye movements required for reading and interpreting various media provide practice for this pattern of attention.

Within the scope of comparative price ads, the current study finds that when regular and discount prices are presented horizontally on the same page, consumers tend to direct their attention first to the information on the left and move their attention to the right. This natural progression from left to right shapes how prices are perceived and evaluated.

Given the inherent left-to-right directionality in attention, we conclude that when the regular price is positioned on the left, it is likely perceived as the starting point of a pricing narrative, with attention naturally progressing to the discount price on the right, which then serves as the endpoint in comparative price advertisements and vice versa.

### 2.3 Reference price judgment and horizontal price locations

As previously discussed, the anchoring and adjustment theory is foundational in our research, explaining how initial information—referred to as anchors—affects subsequent judgments (Tversky and Kahneman, 1974; Epley and Gilovich, 2010; Galinsky and Mussweiler, 2001; LeBoeuf and Shafir, 2006; Suk et al., 2012). This theory has been pivotal in understanding how reference prices are formed, a concept frequently manipulated in various pricing contexts (Ahmetoglu et al., 2014). A reference price is typically perceived by consumers as the ‘normal’ price, whether it be an average market price or a standard undiscounted price. Prevalent market practices shape reference pricing by comparing the advertised price to (1) the price previously charged by the retailer (i.e., regular price), (2) prices charged by competitors within the same industry, and (3) manufacturer-suggested retail prices, which collectively influence consumer perceptions of potential savings and the purchase value (Ahmetoglu et al., 2014; Grewal et al., 1998; Lichtenstein and Bearden, 1989).

In comparative price advertisements, the focus of our study, the theory particularly comes into play as consumers are expected to compared regular and sale prices. Anchoring and adjustment theory provides a crucial mechanism for understanding how consumers estimate reference prices based on the information presented. The horizontal positioning of regular and discount prices plays a critical role due to the universal left-to-right reading pattern observed across various cultures. When prices are displayed horizontally, the price positioned on the left becomes the initial piece of information processed by consumers. This price then serves as an anchor, establishing a cognitive baseline that significantly influences the subsequent formation of reference prices.

Thus, if the regular price is positioned on the left rather than the right, it serves as the primary anchor. This display location typically results in a heightened reference price judgment, as the initial anchor, here the regular price, is generally more than the discount price. This effect is consistent with findings that the first, higher numerical value seen influences perceptions of value and quality more strongly than subsequent lower values, leading to higher overall price assessments (Ahmetoglu et al., 2014; Davis and Bagchi, 2018; Epley and Gilovich, 2010; Hogarth and Einhorn, 1992; Kardes and Kalyanaram, 1992; Suk et al., 2012; Tversky and Kahneman, 1974).

*H1 (main effect)*: Individuals will exhibit a higher reference price judgment when the regular price is located to the left (vs. right) of the discount price.

This hypothesis is based on the directional bias of reading and processing information from left to right, where the first price seen influences the consumer’s internal reference price more strongly. The regular price, when positioned as an initial anchor, sets a higher comparative standard, making subsequent prices seem more favorable or reasonable.

### 2.4 Psychological underlying mechanism: perceived product quality and horizontal price locations

Building upon anchoring and adjustment theory, the previous section discussed how initial information presented in comparative price advertisements influences reference price judgments. We now extend this discussion to explore perceived product quality under the common consumer heuristic that higher prices signify superior quality. This heuristic, supported by theories of causal relationships that guide interpretations and predictions (Kramer et al., 2012; Labroo and Mukhopadhyay, 2009; Van Ooijen et al., 2017; Woolley et al., 2023), highlights the well-documented marketplace bias of price-quality inference (Kardes et al., 2004a,b; Lichtenstein et al., 1993; Rao and Monroe, 1989; Weisstein et al., 2019). Often reinforced by biased sampling from past experiences and media exposure, this belief compels consumers to use price as a primary quality indicator (Haws et al., 2017; Kardes et al., 2004a,b).

In comparative price advertisements, when information is processed from left to right, the price on the left becomes the first-encoded anchor, potentially serving as a reference for quality (Suk et al., 2012; Dogerlioglu-Demir et al., 2023). When the regular price, which is typically higher than the discount price, is positioned on the left, it becomes the first piece of information consumers encounter. This positioning naturally leads to the regular price serving as a primary quality indicator, based on “higher price implies higher quality” heuristic at the outset, biasing subsequent quality assessments. Consequently, this initial encoding not only affects the perception of the offered price promotion but also biases the interpretation of the product as being of higher quality due to its association with a higher price point. This dynamic ensures that, compared to when the discount price is the first-encoded anchor, having the regular price as the first point of engagement significantly elevates inferred quality, reflecting the heuristic that a higher price implies superior quality.

The strategic positioning of regular and discount prices significantly impacts consumers’ quality perceptions due to these inherent biases. In conditions where the regular price is positioned on the left, it is typically processed first because of the prevalent left-to-right reading pattern. This positioning allows the regular price to act as a potent anchor, potentially leading to higher perceived product quality. This effect arises because the regular price, often higher than the discount price, sets a strong initial standard that may elevate consumers’ quality expectations based on the associated price. This dynamic aligns with the notion that briefs about price-quality correlations, once activated, guide how additional product information is encoded, recalled, or deemed diagnostically relevant (Higgins, 1996; Kardes et al., 2004a,b). Formally, we propose:

*H2 (Main Effect)*: Individuals will exhibit a higher perceived product quality when the regular price is located to the left (vs. right) of the discount price.

This framework explains how minor shifts in the horizontal positioning of price displays can have significant impacts on consumer judgments, illustrating how first-encoded price information can steer price-quality inferences by leveraging an existing belief about price-quality relationships. These insights illustrate the integration of consumers’ lay belief with anchoring processes, shaping not only perceptions of price promotion but also perceived overall value and quality, significantly influencing consumer judgments.

Furthermore, we propose a mediation hypothesis to capture the comprehensive impact of these dynamics on consumer valuation. Extensive research shows that when consumers perceive a product to be of higher quality, they not only form stronger purchase intentions but also become more inclined to accept higher prices for that product (Lichtenstein and Bearden, 1989; Weisstein et al., 2019). From an anchoring perspective, once a higher regular price on the left triggers the “price implies quality” heuristic, consumers adjust their subsequent willingness to pay and internal reference price upward, effectively reinforcing the influence of the first price seen (Epley and Gilovich, 2010). In other words, the initial perception of quality catalyzes a cascading effect on broader price evaluations, guiding how consumers judge the fairness and overall value of the offer (Kahneman et al., 1982; Xia et al., 2004).

We formally posit the following mediation hypothesis:

*H3 (Mediation)*: Perceived quality will mediate the effect of regular and discount price locations on reference price judgment.

This hypothesis suggests that the initial placement of price information not only shapes immediate quality perceptions but also systematically affects consumers’ long-term valuation of the product. By enhancing perceived quality, the left-positioned regular price influences consumers’ internal benchmarks for determining what constitutes a “fair” or “reasonable” price, thereby elevating their reference price judgments. This mediational pathway provides deeper insight into the psychological mechanisms by which strategic horizontal price positioning can influence both consumers’ qualitative assessments (e.g., quality inferences) and quantitative judgments (e.g., reference prices).

## 3 Studies

### 3.1 Study 1A: price location effect

Study 1 aims to investigate the price location effect—the influence of positioning regular and discount prices on perceived reference prices in comparative price advertisements. It is hypothesized that participants will report a higher reference price when the regular price is positioned on the left relative to the discount price on the right, versus the reverse arrangement (H1).

#### 3.1.1 Material and method

##### 3.1.1.1 Sample size

The required sample size was validated using G*Power analysis, a recognized tool for determining sufficient sample sizes in experimental research. Utilizing G*Power version 3.1.9.7 (Kang, 2021; Narayanan, 2024), we set parameters for a power of 0.8, an expected effect size of Cohen’s *f* = 0.25 (η_p_^2^ = 0.06), *α* = 0.05, and number of groups = 3. The analysis indicated that a sample size of 159 participants was necessary.

##### 3.1.1.2 Participants and design

A total of 214 online participants from the U.S. (35.0% female, M_age_ = 30.8, SD = 9.27) were recruited via Amazon Mechanical Turk (MTurk) for a one-way between-subjects experimental design. The independent variable was price display locations (regular price left vs. discount price left vs. no promotion/regular price only). Participants were randomly assigned to one of the three conditions and received a monetary reward of $0.50 for their participation, consistent with standard practices in behavioral research.

##### 3.1.1.3 Methodology for sampling and survey distribution

To ensure consistent data collection across Studies 1A, 1B, and 2, uniform methodologies were employed using two primary platforms. First, survey questionnaires were developed on the Qualtrics platform, enabling the random distribution of distinct versions of the experimental materials. This minimized selection bias and ensured methodological rigor and reliability in the data collection (Campbell and Stanley, 2015; Shadish et al., 2002). Second, MTurk served a platform to access a diverse and representative sample of the U.S. population, which aligns with our target populations for hypothesis testing. Given that our primary hypotheses do not anticipate demographic disparities (e.g., age or gender), utilizing a platform like MTurk is ideal for capturing consumer responses in a natural setting, enabling the application of our findings to real-market examinations. More importantly, MTurk is highly regarded among behavioral researchers for its reliable, high-quality, and demographically diverse respondent pool (Moss, 2020; Moss et al., 2023; Septianto et al., 2020), reflecting the financial situation of the general U.S. population (Moss et al., 2023). Its ability to mirror national demographic diversity is invaluable for ensuring representativeness in behavioral studies (Moss, 2020). This feature makes MTurk particularly suitable for price-related research questions and has been proven to ensure data quality in advertising research (Berry et al., 2022; Kees et al., 2017). Utilizing MTurk helps us to generalize our findings effectively across a typical U.S. consumer base, providing robust market insights without demographic constraints.

To ensure the reliability of responses, participants were prescreened based on two criteria: (1) a HIT approval rate above 90%, ensuring high-quality responses as recommended by Hauser and Schwarz (2016); and (2) U.S. residency, aged 18 years and older, required to control for market-specific price variations and maintain uniformity in reference price evaluations across the sample.

##### 3.1.1.4 Stimuli and procedures

The advertisement featured a product image and a brief description. In the no promotion condition, only the regular price was displayed. In the other conditions, the regular ($22.50) and discount prices ($15.75) were aligned horizontally, representing a discount rate of about 30%, considered moderate in depth (Biswas et al., 2013; Kim and Jang, 2022; Grewal et al., 1998; Lichtenstein et al., 1991). The regular price was positioned either to the left or the right of the discount price, varying by condition (see Appendix A).

As previously mentioned, the study employed an online survey questionnaire developed on the Qualtrics platform. The Qualtrics link to the survey was distributed through the MTurk online platform, with all procedures conducted online. Upon accessing the survey link, participants were provided with general instructions and confirmed their participation by agreeing to the consent form.

Participants viewed one advertisement depending on their experimental condition and then estimated the internal reference price for the product using an open-ended format (“How much do you think is a fair price for this advertised product in the market?”) (Lichtenstein and Bearden, 1989). Subsequently, demographic information was collected from the respondents. Demographic information was subsequently collected. Among the 213 participants, 139 (65.3%) identified as male and 74 (34.7%) identified as female. The age distribution was as follows: 28.2% under 24 years old, 44.6% aged 25–34, 16.0% aged 35–44, 8.5% aged 45–54, and 2.8% over 55 years old. For detailed demographic statistics of the participants, please see Table 1.

**Table 1:** Demographic statistics of participants across all studies.

Studies	Study 1a		Study 1b		Study 2	
Sample size	213		101		121	
	*n*	%	*n*	%	*n*	%
Gender						
Male	139	65.3	59	58.4	83	68.6
Female	74	34.7	42	41.6	38	31.4
Age in years						
Less than 24	60	28.2	4	4.0	10	8.3
25 to 34	95	44.6	46	45.5	48	39.6
35 to 44	34	16.0	33	32.7	39	32.2
45 to 54	18	8.5	11	10.9	14	11.6
Over 55	6	2.8	7	6.9	10	8.3
Level of education						
High school/Technical school			11	10.9	33	27.3
Undergraduate/Associate degree			72	71.3	68	56.2
Postgraduate degree			18	17.8	20	16.5
Annual household income						
Less than $25,000			6	5.9		
$25,000 ~ $49,999			20	19.8		
$50,000 ~ $74,999			39	38.6		
$75,000 ~ $99,999			31	30.7		
$100,000 or more			5	5.0		

Notably, In line with previous research, we define the internal reference price primarily as the fair market price, which is part of a broader multidimensional construct. This construct captures not only the perceived normal or average market price but also a range of acceptable prices, from the highest participants are willing to pay to the lowest they consider acceptable in the market (reservation price and minimum market price) (Thaler, 1985; Urbany et al., 1988). By focusing on the fair market price, we closely align our operational definition with well-established scholarly frameworks.

#### 3.1.2 Results

To confirmed the proposed hypothesis, one-way ANOVA was conducted to examine the effect of price display locations on internal reference price judgments, specifically fair market price estimations. The analysis revealed a significant main effect for price display locations (*F*(2, 207) = 14.40, *p* < 0.001). Specifically, participants in the no promotion condition estimated a higher fair market price (*M* = 22.24, *SD* = 5.79) compared to those in the regular price left condition (*M* = 18.89, *SD* = 5.39; *F*(1, 207) = 14.03, *p* < 0.001, *d* = 0.69) and the discount price left condition (*M* = 17.35, *SD* = 3.48; *F*(1, 207) = 30.54, *p* < 0.001, *d* = 1.01). These findings suggest that price promotions generally lower the internal reference prices individuals assign to the promoted product.

More importantly, detailed comparison between the two price promotion conditions varying price locations revealed that the estimated fair price was notably higher when the regular price was positioned on the left as opposed to when the discount price was on the left (*M*_regular price left_ = 18.89 vs. *M*_discount price left_ = 17.35; *F*(1,207) = 4.22, *p* = 0.041, *d* = 0.32). This finding supports Hypothesis 1, indicating that the adverse impact of price promotions on reference price judgments can be mitigated by strategically aligning the regular price to the left of the discount price. Additionally, the results remained consistent and significant even after including demographic variables as covariates.

#### 3.1.3 Discussion

Study 1A provides initial evidence in support of our hypothesis regarding the price location effect. We hypothesize that this effect arises from anchoring and adjustment heuristics, whereby individuals anchor on the price information presented on the right and use it to infer product quality and subsequently reference price. If this underlying mechanism holds true, the price location effect might be attenuated by providing explicit information about product quality.

The previous literature on heuristics (Gunasti and Ross, 2010; Hsee et al., 2009; Kardes et al., 2004a,b; Maheswaran et al., 1992) indicates that individuals often depend on numerical information, especially in the absence of clear and relevant data for decision-making. This tendency can be countered by providing explicit, relevant information. Building on this insight, our study suggests that introducing explicit quality indicators, such as certification marks, can reduce reliance on price positioning by offering clearer cues for assessing product quality (Gunasti and Ross, 2010). These indicators allow consumers to use established knowledge to evaluate quality, thus potentially diminishing the impact of initial price presentations on perceived product quality and reference price judgments. Study 1B is designed to rigorously test this mechanism by examining whether the presence of certification marks can mitigate or nullify the price location effect.

### 3.2 Study 1B: replication and boundary condition of price location effect

Study 1B aims to replicate the price location effect observed in Study 1A and test the proposed underlying mechanism by introducing a boundary condition—the presence of explicit quality indicators, specifically quality certification marks. We hypothesize that in the absence of these marks, participants will rely more heavily on price information presented on the right when forming reference price judgments. In contrast, the presence of certification marks is expected to neutralize the price location effect, thus affirming the role of anchoring and adjustment heuristics in this context.

To enhance the generalizability of our findings, Study 1B features a different experimental setup: (1) it presents a screenshot simulating an online shopping environment to more closely reflect real-world shopping scenarios, and (2) it employs a different product category, a dietary supplement, than the Sony headphones used in Study 1A. The selection of dietary supplements for Study 1B aims to test the robustness of our hypotheses across varied consumer goods categories.

#### 3.2.1 Method

##### 3.2.1.1 Sample size

As with Study 1A, the required sample size for Study 1B was determined using G*Power analysis (Kang, 2021; Narayanan, 2024). Adjustments were made to accommodate the number of groups, set at 4, consistent with the requirements from Study 1A. This analysis suggested that a sample size of 101 participants was necessary.

##### 3.2.1.2 Participants and design

This study employed a 2 (price display location: regular price left vs. discount price left) X 2 (quality certification mark: presence vs. absence) between-subject design manipulating both variables. A total of 101 U.S. participants were recruited via MTurk. The demographic breakdown included 59 males (58.4%) and 42 females (41.6%), with a mean age of 35.97 years (SD = 9.58). Participants’ ages ranged from 20 to 67 years. Educational levels were distributed as follows: 11 participants (10.9%) had high school or technical school education, 72 (71.3%) had an undergraduate or associate degree, and 18 (17.8%) held postgraduate degrees. Regarding annual household income: 5.9% earned below $25,000; 19.8% between $25,000 and $49,999; 38.6% between $50,000 and $74,999; 30.7% between $75,000 and $99,999; and 5.0% above $100,000. Detailed demographic statistics can be found in Table 1. Participants were randomly assigned to one of four experimental conditions, thereby ensuring varied exposure to the different experimental stimuli. Each participant received a monetary reward of $0.50 for their participation.

##### 3.2.1.3 Stimuli and procedures

The stimuli were designed to resemble a typical online shopping environment, specifically modeling the layout commonly used by platforms like Amazon.com. Each stimulus included a product image, a brief description, brand name, price information, and other relevant details. The price location manipulation was consistent with Study 1A, featuring a dietary supplement with a regular price of US $36.5 and a discount price of US $25.55. In the condition with quality certification marks, these marks were prominently displayed above the product description. In the condition without these marks, only the product descriptions were shown (see Appendix B for details).

Procedures mirrored those of Study 1A. After viewing the online shopping page, participants were requested to state their perceived fair market price for the product, which reflected their internal reference price. This response was captured using an open-ended format (“How much do you think is a fair price for this advertised product in the market?”) (Lichtenstein and Bearden, 1989; Thaler, 1985; Urbany et al., 1988). Finally, demographic information was collected from all respondents.

#### 3.2.2 Results

One-way ANOVA was conducted to examine the dynamic interaction between price display locations and the presence of quality certification marks on internal reference price judgments, focusing on fair market price estimations. Consistent with our expectations, the analysis revealed a significant interaction effect between price display locations and quality certification marks (*F*(1, 101) = 3.86, *p* = 0.052, *d* = 0.32). Without quality certification marks, replicating the context of Study 1A, the estimated fair price was significantly higher when the regular price was positioned on the left rather than the discount price (*M*_regular price left_ = 26.31, *SD* = 8.22 vs. *M*_discount price left_ = 21.02, *SD* = 9.63; *F*(1,101) = 5.39, *p* = 0.03, *d* = 0.68). However, this effect was neutralized in the presence of quality certification marks (*M*_regular price left_ = 27.19, *SD* = 5.86 vs. *M*_discount price left_ = 28.27, *SD* = 8.55; *F*(1,101) = 0.22, *p* = 0.64, *d* = 0.15; not significant). Additionally, the results remained consistent and significant even after controlling for demographic variables and the shopping platform as covariates. This result aligns with our theorizing that explicit quality indicators can mitigate the influence of initial price positioning on reference price judgments, thereby supporting the validity of anchoring and adjustment heuristics as a foundational theory for our Hypothesis 1.

#### 3.2.3 Discussion

The findings from Study 1B robustly support Hypothesis 1 and our theoretical rationale, demonstrating that the price location effect is indeed rooted in anchoring and adjustment heuristics. Consistent with our theoretical framework, this effect was reliably replicated under conditions that lacked direct, objective indicators of product quality, where judgments about quality are inherently subjective and uncertain. Conversely, the introduction of explicit quality indicators, such as certification marks, effectively neutralized the price location effect. This suggests that participants did not rely on price positioning—specifically, prices presented on the right—as a heuristic cue when definitive quality information was available. Instead, they used the quality certification marks as the primary basis for evaluating product quality and judging reference price. This shift highlights the crucial role of explicit quality cues in moderating the influence of price display order on consumer perceptions and decision-making, affirming the prevalent role of heuristics in such contexts (Gunasti and Ross, 2010; Hsee et al., 2009; Kardes et al., 2004a,b; Maheswaran et al., 1992). While these findings provide compelling evidence, further exploration into the direct mechanisms involved is essential. To address this, Study 2 is introduced to deepen our understanding.

### 3.3 Study 2: mediating role of perceived quality

The primary goal of Study 2 is to examine how the locations of regular and discount prices influence perceived quality in comparative price advertising (H2) and whether perceived quality mediates the relationship between price display locations and reference price (H3). This investigation is designed to provide more direct evidence and clarify the mechanisms underlying the price location effect. Additionally, to test the generalizability of the price location effect (H1), Study 2 employs a travel bag as the stimulus, which diverges from the product types used in Studies 1A and 1B—Sony headphones and dietary supplements, respectively. This variation helps to establish the robustness of the findings across different contexts.

#### 3.3.1 Method

##### 3.3.1.1 Sample size

As with previous studies, the necessary sample size for Study 2 was validated using G*Power analysis (Kang, 2021; Narayanan, 2024), requiring 106 participants.

##### 3.3.1.2 Participants and design

Study 2 engaged 121 participants from the U.S. via MTurk, consisting of 83 males (68.6%) and 38 females (31.4%), with a mean age of 36.6 years (SD = 8.83) and an age range of 19 to 67 years. Educational levels were 27.3% high school/technical or less, 56.2% undergraduate/associate degree, and 16.5% postgraduate degree. Detailed demographics are in Table 1. Participants were randomly assigned to either one of the price display location conditions; the regular price left condition or the discount price left condition. Each participant was compensated with $0.50 for their participation. The general manipulation and procedure mirrored those of Study 1A, with three key differences: the exclusion of a no-promotion condition, the inclusion of items to measure perceived product quality as a potential mediator, and the use of a different stimulus—a Samsonite travel bag—to enhance generalizability.

##### 3.3.1.3 Stimuli and procedures

The travel bag was presented with a regular price of US $280 and a discount price of US $190 (see Appendix C). Following their exposure to the advertisement, participants were requested to determine a fair market price, similar to the method used in Study 1. Perceived quality was then assessed with two items (“The product has good quality,” “The product seems qualified”) on a 7-point scale (Cronbach *α* = 0.91, *M* = 5.68, *SD* = 1.05). Finally, demographic information was collected from the respondents.

#### 3.3.2 Results

##### 3.3.2.1 Reference price

In line with our expectations, a significant main effect of price location on the reference price was observed. The fair market price reported for the advertised product was higher when the regular price was displayed on the left as opposed to the discount price (*M*_regular price left_ = 239.76, *SD* = 48.14 vs. *M*_discount price left_ = 215.08, *SD* = 66.65; *F*(1,119) = 5.37, *p* = 0.022, *d* = 0.42). Thus, this result supported H1.

##### 3.3.2.2 Perceived quality

The effect of price location on perceived product quality was also significant. The product was perceived as higher quality when the regular price was displayed on the left rather than the discount price (*M*_regular price left_ = 5.90, *SD* = 0.85 vs. *M*_discount price left_ = 5.56, *SD* = 0.97; *F*(1,119) = 4.00, *p* = 0.048, *d* = 0.37). Thus, this result supported H2.

##### 3.3.2.3 Mediation test

To explore the role of perceived product quality as a mediator in the relationship between price display location and reference price judgment, a mediation analysis was conducted using the PROCESS macro for SPSS (Hayes, 2017, Model 4, 5,000 bootstrap samples) with 95% confidence intervals (CIs). This analysis followed the approach recommended by Zhao et al. (2010), with price location (coded as −1 = discount price left, 1 = regular price left) serving as the independent variable, perceived product quality as the mediator, and reference price as the dependent variable.

**Figure 1:**
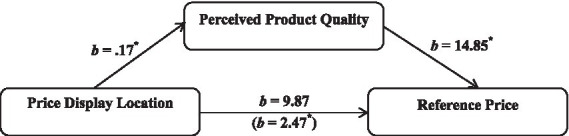
Research model and mediation analysis. ^*^ *p* < 0.05, ^**^ *p* < 0.01.

The analysis indicated a significant influence of price display location on perceived product quality (*b* = 0.17, *t* = 2.09, *p* = 0.038). Perceived product quality in turn significantly impacted reference price judgment (*b* = 14.85, *t* = 2.47, *p* = 0.015). The mediation analysis further highlighted a significant indirect effect of price display location on reference price judgment through the mediator, perceived product quality (indirect effect = 2.47, 95% CI [0.187, 6.935]). Meanwhile, the direct effect of price display location on reference price judgment was non-significant (*b* = 9.87, *t* = 1.86, *p* = 0.07), confirming the full mediation model suggested by perceived product quality (Zhao et al., 2010). Additionally, the robustness of these findings was maintained even after controlling for demographic variables as covariates. These findings validate the mediation hypothesis and support H3 (see Figure 1).

#### 3.3.3 Discussion

Study 2 tested H1 to H3 within a comparative price advertising context, where regular and discount prices were horizontally aligned on the same page. We observed that when the regular price was presented on the left and the discount price on the right, participants reported higher reference prices and perceived product quality. This study also confirmed that perceived product quality mediated the relationship between price display locations and reference price judgments. Consistent with our hypotheses, the findings from Studies 1A, 1B and 2 indicate that consumer responses to price promotions are more favorable when the regular price is positioned to the left of the discount price.

## 4 General discussion

### 4.1 Conclusion

This study underscores the influence of specific price display locations—regular prices positioned to the left of discount prices—on the formation of higher internal reference prices in promotional materials. Our findings are consistent across different products; headphones and dietary supplement in Study 1 and travel bags in Study 2, highlighting the robustness of the location effect. Importantly, we confirmed that the impact of price display locations on reference price judgment is mediated through acquisition values, specifically perceived product quality.

#### 4.1.1 Theoretical contributions

Building on Thaler’s (1985) conceptual framework, prior studies have predominantly focused on how price information placement affects transaction values of a promotion such as perceived savings and deal attractiveness (Bagchi and Davis, 2012; Biswas et al., 2013; DelVecchio et al., 2009). Our research extends these insights by showing that display locations also significantly influence acquisition value, notably affecting the product quality perceived by consumers in comparative price advertisements.

We utilized the anchoring and adjustment theory as a foundational framework to illustrate how internal reference prices are shaped by the price information presented in promotions. Unlike previous research that typically employed a single stimulus variant as an anchor in marketing contexts (i.e., Barbera et al., 2018), our study presented the same price information in both experimental conditions but altered the sequence. This introduction of horizontal price display as a novel factor that shifts attention and serves as a potential anchor, this study enriches our understanding of how long-term consumer perceptions and decisions, such as reference price judgment, are formed.

Further, by extending previous reference price research (e.g., Ahmetoglu et al., 2014; Grewal et al., 1998; Lichtenstein and Bearden, 1989; Weisstein et al., 2019), and introducing reference price and perceived product quality as novel outcome variables within this underexplored context, our research significantly enhances the field of consumer psychology, particularly in comparative price advertisements. This offers new insights into the psychological mechanisms that underlie consumer responses, thereby enriching the theoretical discourse on how environmental and informational cues influence the formation of consumers’ reference prices.

#### 4.1.2 Managerial implications

Our study offers actionable guidance for marketers. Our observation demonstrates that a subtle adjustment in the display locations of price information, specifically positioning the regular price before the discount price in horizontal displays, can enhance perceived product quality and mitigate the negative impacts of price promotions, such as perceptions of reduced quality and potential damage to brand image. These insights encourage more effective design of price promotions, aimed at optimizing consumer perceptions and judgments, thus improving overall consumer satisfaction and brand perception.

In summary, this discussion links the strategic positioning of price information to practical outcomes, enabling marketing practitioners to develop pricing strategies that enhance perceived savings and quality, positively influence consumer reference price judgment, and ultimately increase consumer satisfaction.

### 4.2 Limitations and future research

This study investigated the impact of moderate price discounts, approximately 30%, as prior research indicates that price location effects are particularly salient at this discount level (Kim and Jang, 2022; Biswas et al., 2013). However, the intensity of these effects might vary with different discount depths. Both shallower and deeper than those tested here. Future research could explore the moderating role of discount depth to ascertain the boundaries and generalizability of our findings.

Additionally, future studies might examine the differences between vertical and horizontal displays of price information. Prior research has documented distinct impacts depending on whether prices are presented vertically or horizontally (Choi and Coulter, 2012; Coulter and Norberg, 2009). Since consumers typically scan prices in a top-to-bottom and left-to-right sequence, price information displayed on the left or at the top of an advertisement might have a more pronounced anchoring effect.

A significant aspect of this study is its focus on the potential long-term effects of price display strategies, using reference price as a dependent variable. Prior research, such as that by Chandrashekaran and Grewal (2006), has typically measured reference prices several days after initial exposure to the promotional context, indicating that reference price formation is influenced by information that has been previously seen and encoded (i.e., Kalwani et al., 1990; Rajendran and Tellis, 1994). Our findings highlight that the horizontal positioning of price information can significantly alter the information encoded in consumers’ minds, potentially leading to lasting judgments. However, our study did not replicate the precise methods used in earlier research to measure these effects. Future research should directly investigate these dynamics to provide a more comprehensive understanding of how promotional framing influences consumer decisions over time.

Despite rigorous hypothesis testing, the study faces methodological constraints that limit the generalizability of the findings, including a relatively small sample size which may not fully capture demographic diversity or represent underrepresented subgroups adequately. Furthermore, the study did not consider the potential influence of brand perception, particularly with well-known brands like Sony, which could impact consumer decision-making. Future research should utilize larger, more diverse samples and examine the role of brand perception in the proposed price display strategies to gain deeper insights into their collective effects on consumer behavior.

## Data Availability

The raw data supporting the conclusions of this article will be made available by the authors, without undue reservation.

## References

[ref1] Ahmetoglu G. Furnham A. Fagan P. (2014). Pricing practices: a critical review of their effects on consumer perceptions and behaviour. J. Retail. Consum. Serv. 21, 696–707. doi: 10.1016/j.jretconser.2014.04.013

[ref2] Bagchi R. Davis D. F. (2012). $29 for 70 items or 70 items for $29? How presentation order affects package perceptions. J. Consum. Res. 39, 62–73. doi: 10.1086/661893

[ref3] Barbera M. Northey G. Septianto F. Spanjaard D. (2018). Those prices are HOT! How temperature-related visual cues anchor expectations of price and value. J. Retail. Consum. Serv. 44, 178–181. doi: 10.1016/j.jretconser.2018.06.012

[ref4] Berry C. Kees J. Burton S. (2022). Drivers of data quality in advertising research: differences across MTurk and professional panel samples. J. Advert. 51, 515–529. doi: 10.1080/00913367.2022.2079026

[ref5] Biswas A. Bhowmick S. Guha A. Grewal D. (2013). Consumer evaluation of sale prices: role of the subtraction principle. J. Mark. 77, 49–66. doi: 10.1509/jm.12.0052

[ref6] Campbell D. T. Stanley J. C. (2015). Experimental and quasi-experimental designs for research: Ravenio books.

[ref7] Chandrashekaran R. (2004). The influence of redundant comparison prices and other price presentation formats on consumers' evaluations and purchase intentions. J. Retail. 80, 53–66. doi: 10.1016/j.jretai.2004.01.004

[ref8] Chandrashekaran R. Grewal D. (2006). Anchoring effects of advertised reference price and sale price: the moderating role of saving presentation format. J. Bus. Res. 59, 1063–1071. doi: 10.1016/j.jbusres.2006.06.006

[ref9] Chatterjee A. Southwood M. H. Basilico D. (1999). Verbs, events and spatial representations. Neuropsychologia 37, 395–402. doi: 10.1016/S0028-3932(98)00108-0, PMID: 10215086

[ref10] Choi P. Coulter K. S. (2012). It's not all relative: the effects of mental and physical positioning of comparative prices on absolute versus relative discount assessment. J. Retail. 88, 512–527. doi: 10.1016/j.jretai.2012.04.001

[ref11] Christman S. Pinger K. (1997). Lateral biases in aesthetic preferences: pictorial dimensions and neural mechanisms. Laterality 2, 155–175. doi: 10.1080/713754266 15513061

[ref12] Competera. (2023). Discount pricing strategy: definition & example. Available at: https://competera.ai/resources/articles/discount-pricing-strategy-definition-example

[ref13] Coulter K. S. Norberg P. A. (2009). The effects of physical distance between regular and sale prices on numerical difference perceptions. J. Consum. Psychol. 19, 144–157. doi: 10.1016/j.jcps.2009.02.008

[ref14] Davis D. F. Bagchi R. (2018). How evaluations of multiple percentage price changes are influenced by presentation mode and percentage ordering: the role of anchoring and surprise. J. Mark. Res. 55, 655–666. doi: 10.1177/0022243718808554

[ref15] Della Bitta A. J. Monroe K. B. McGinnis J. M. (1981). Consumer perceptions of comparative price advertisements. J. Mark. Res. 18, 416–427. doi: 10.1177/002224378101800402

[ref16] DelVecchio D. Lakshmanan A. Krishnan H. S. (2009). The effects of discount location and frame on consumers' price estimates. J. Retail. 85, 336–346. doi: 10.1016/j.jretai.2009.05.010

[ref17] Dogerlioglu-Demir K. Koçaş C. Cavdar Aksoy N. (2023). The role of presentation order in consumer choice: the abrupt disparity effect. Mark. Lett. 34, 251–268. doi: 10.1007/s11002-022-09643-6

[ref18] Einhorn H. J. Hogarth R. M. (1986). Decision making under ambiguity. J. Bus. 59, S225–S250. doi: 10.1086/296364

[ref19] Epley N. Gilovich T. (2006). The anchoring-and-adjustment heuristic: why the adjustments are insufficient. Psychol. Sci. 17, 311–318. doi: 10.1111/j.1467-9280.2006.01704.x, PMID: 16623688

[ref20] Epley N. Gilovich T. (2010). Anchoring unbound. J. Consum. Psychol. 20, 20–24. doi: 10.1016/j.jcps.2009.12.005

[ref21] Galinsky A. D. Mussweiler T. (2001). First offers as anchors: the role of perspective-taking and negotiator focus. J. Pers. Soc. Psychol. 81, 657–669. doi: 10.1037/0022-3514.81.4.657, PMID: 11642352

[ref22] Graciola A. P. De Toni D. de Lima V. Z. Milan G. S. (2018). Does price sensitivity and price level influence store price image and repurchase intention in retail markets? J. Retail. Consum. Serv. 44, 201–213. doi: 10.1016/j.jretconser.2018.06.014

[ref23] Grewal D. Monroe K. B. Krishnan R. (1998). The effects of price-comparison advertising on buyers’ perceptions of acquisition value, transaction value, and behavioral intentions. J. Mark. 62, 46–59. doi: 10.1177/002224299806200204, PMID: 39913176

[ref24] Gunasti K. Ross W. T. (2010). How and when alphanumeric brand names affect consumer preferences. J. Mark. Res. 47, 1177–1192. doi: 10.1509/jmkr.47.6.1177

[ref25] Hauser D. J. Schwarz N. (2016). Attentive Turkers: MTurk participants perform better on online attention checks than do subject pool participants. Behav. Res. Methods 48, 400–407. doi: 10.3758/s13428-015-0578-z, PMID: 25761395

[ref26] Haws K. L. Reczek R. W. Sample K. L. (2017). Healthy diets make empty wallets: the healthy= expensive intuition. J. Consum. Res. 43, ucw078–ucw1007. doi: 10.1093/jcr/ucw078

[ref27] Hayes A. F. (2017). Introduction to mediation, moderation, and conditional process analysis: A regression-based approach. New York, NY: The Guilford Press.

[ref28] Higgins E. T. (1996). Knowledge activation: accessibility, applicability, and salience in Social psychology: handbook of basic principles. eds. Higgins E. T. Kruglanski A. W., (New York, NY: Guilford Press). 133–68.

[ref29] Hogarth R. M. Einhorn H. J. (1992). Order effects in belief updating: the belief-adjustment model. Cogn. Psychol. 24, 1–55. doi: 10.1016/0010-0285(92)90002-J

[ref30] Hsee C. K. Yang Y. Gu Y. Chen J. (2009). Specification seeking: how product specifications influence consumer preference. J. Consum. Res. 35, 952–966. doi: 10.1086/593947

[ref31] Jang J. M. Park E. Y. (2020). Location does matter: the effect of display locations of regular price and sale price on consumers’ responses in comparative price advertising. Int. J. Advert. 39, 1059–1085. doi: 10.1080/02650487.2019.1687233

[ref32] Kahneman D. Slovic P. Tversky A. (1982). Judgment under uncertainty: Heuristics and biases. Cambridge, MA: Cambridge University Press. 10.1126/science.185.4157.1124 17835457

[ref33] Kalwani M. U. Yim C. K. Rinne H. J. Sugita Y. (1990). A price expectation model of consumer brand choice. J. Mark. Res. 27, 251–262. doi: 10.1177/002224379002700301

[ref34] Kalyanaram G. Winer R. S. (2022). Behavioral response to price: data-based insights and future research for retailing. J. Retail. 98, 46–70. doi: 10.1016/j.jretai.2022.02.009

[ref35] Kang H. (2021). Sample size determination and power analysis using the G* power software. J. Educ. Eval. Health Prof. 18:17. doi: 10.3352/jeehp.2021.18.17, PMID: 34325496 PMC8441096

[ref36] Kardes F. R. Cronley M. L. Kellaris J. J. Posavac S. S. (2004a). The role of selective information processing in price-quality inference. J. Consum. Res. 31, 368–374. doi: 10.1086/422115

[ref37] Kardes F. R. Kalyanaram G. (1992). Order-of-entry effects on consumer memory and judgment: an information integration perspective. J. Mark. Res. 29, 343–357. doi: 10.1177/002224379202900305

[ref38] Kardes F. R. Posavac S. S. Cronley M. L. (2004b). Consumer inference: a review of processes, bases, and judgment contexts. J. Consum. Psychol. 14, 230–256. doi: 10.1207/s15327663jcp1403_6

[ref39] Kees J. Berry C. Burton S. Sheehan K. (2017). An analysis of data quality: professional panels, student subject pools, and Amazon’s mechanical Turk. J. Advert. 46, 141–155. doi: 10.1080/00913367.2016.1269304

[ref40] Kim H. Jang J. M. (2022). Disadvantages of red: the color congruence effect in comparative price advertising. Front. Psychol. 13:1019163. doi: 10.3389/fpsyg.2022.1019163, PMID: 36467187 PMC9712978

[ref41] Kramer T. Irmak C. Block L. G. Ilyuk V. (2012). The effect of a no-pain, no-gain lay theory on product efficacy perceptions. Mark. Lett. 23, 517–529. doi: 10.1007/s11002-012-9165-6

[ref42] Labroo A. A. Mukhopadhyay A. (2009). Lay theories of emotion transience and the search for happiness: a fresh perspective on affect regulation. J. Consum. Res. 36, 242–254. doi: 10.1086/597159

[ref43] LeBoeuf R. A. Shafir E. (2006). The long and short of it: physical anchoring effects. J. Behav. Decis. Mak. 19, 393–406. doi: 10.1002/bdm.535

[ref44] Lichtenstein D. R. Bearden W. O. (1989). Contextual influences on perceptions of merchant-supplied reference prices. J. Consum. Res. 16, 55–66. doi: 10.1086/209193

[ref45] Lichtenstein D. R. Burton S. Karson E. J. (1991). The effects of semantic cues on consumer perceptions of reference price ads. J. Consum. Res. 18, 380–391. doi: 10.1086/209267

[ref46] Lichtenstein D. R. Ridgway N. M. Netemeyer R. G. (1993). Price perceptions and consumer shopping behavior: a field study. J. Mark. Res. 30, 234–245. doi: 10.1177/002224379303000208

[ref47] Maass A. Russo A. (2003). Directional bias in the mental representation of spatial events: nature or culture? Psychol. Sci. 14, 296–301. doi: 10.1111/1467-9280.14421 12807400

[ref48] Maheswaran D. Mackie D. M. Chaiken S. (1992). Brand name as a heuristic cue: the effects of task importance and expectancy confirmation on consumer judgments. J. Consum. Psychol. 1, 317–336. doi: 10.1016/S1057-7408(08)80058-7

[ref49] Mastercard Services. (2024). Retail industry trends 2024. Available at: https://www.mastercardservices.com/en/industries/retail/insights/retail-industry-trends-2024

[ref50] Mendonça R. Garrido M. V. Semin G. R. (2022). Two cultural processing asymmetries drive spatial attention. Cogn. Sci. 46:e13185. doi: 10.1111/cogs.13185, PMID: 35973007

[ref51] Moss A. (2020). Demographics of people on Amazon mechanical Turk. CloudResearch. Retrieved August 10, 2024. Available at: https://www.cloudresearch.com/resources/blog/who-uses-amazon-mturk-2020-demographics/

[ref52] Moss A. J. Rosenzweig C. Robinson J. Jaffe S. N. Litman L. (2023). Is it ethical to use mechanical Turk for behavioral research? Relevant data from a representative survey of MTurk participants and wages. Behav. Res. Methods 55, 4048–4067. doi: 10.3758/s13428-022-02005-0, PMID: 37217711

[ref53] Narayanan P. (2024). Against the green Schema: how gen-AI negatively impacts green influencer posts. Psychol. Mark. 1–17. doi: 10.1002/mar.22159, PMID: 39912375

[ref54] Oppenheimer D. M. LeBoeuf R. A. Brewer N. T. (2008). Anchors aweigh: a demonstration of cross-modality anchoring and magnitude priming. Cognition 106, 13–26. doi: 10.1016/j.cognition.2006.12.008, PMID: 17286970

[ref55] Ouellet M. Santiago J. Funes M. J. Lupiáñez J. (2010). Thinking about the future moves attention to the right. J. Exp. Psychol. Hum. Percept. Perform. 10, 72–82. doi: 10.1037/a0017162, PMID: 20121292

[ref56] Palmeira M. M. Srivastava J. (2013). Free offer ≠ cheap product: a selective accessibility account on the valuation of free offers. J. Consum. Res. 40, 644–656. doi: 10.1086/671565

[ref57] Rajendran K. N. Tellis G. J. (1994). Contextual and temporal components of reference price. J. Mark. 58, 22–34. doi: 10.1177/002224299405800102

[ref58] Rao A. R. Monroe K. B. (1989). The effect of price, brand name, and store name on buyers' perceptions of product quality: an integrative review. J. Mark. Res. 28, 307–357. doi: 10.2307/3172866, PMID: 38532229

[ref59] Santiago J. Lupiáñez J. Pérez E. Funes M. J. (2007). Time (also) flies from left to right. Psychon. Bull. Rev. 14, 512–516. doi: 10.3758/BF03194099, PMID: 17874598

[ref60] Santiago J. Román A. Ouellet M. Rodríguez N. Pérez-Azor P. (2008). In hindsight, life flows from left to right. Psychol. Res. 74, 59–70. doi: 10.1007/s00426-008-0220-0, PMID: 19104828

[ref61] Septianto F. Kemper J. A. Choi J. J. (2020). The power of beauty? The interactive effects of awe and online reviews on purchase intentions. J. Retail. Consum. Serv. 54:102066. doi: 10.1016/j.jretconser.2020.102066

[ref62] Shadish W. R. Cook T. D. Campbell D. T. (2002). Experimental and quasi-experimental designs for generalized causal inference. Boston, MA: Houghton Mifflin.

[ref63] Spalek T. M. Hammad S. (2005). The left-to-right bias in inhibition of return is due to the direction of reading. Psychol. Sci. 16, 15–18. doi: 10.1111/j.0956-7976.2005.00774.x, PMID: 15660846

[ref64] Suk K. Lee J. Lichtenstein D. R. (2012). The influence of price presentation order on consumer choice. J. Mark. Res. 49, 708–717. doi: 10.1509/jmr.11.0309

[ref65] Thaler R. (1985). Mental accounting and consumer choice. Mark. Sci. 4, 199–214. doi: 10.1287/mksc.4.3.199, PMID: 19642375

[ref66] Tversky A. Kahneman D. (1974). Judgment under uncertainty: heuristics and biases. Science 185, 1124–1131. doi: 10.1126/science.185.4157.1124, PMID: 17835457

[ref67] Urbany J. E. Bearden W. O. Weilbaker D. C. (1988). The effect of plausible and exaggerated reference prices on consumer perceptions and price search. J. Consum. Res. 15, 95–110. doi: 10.1086/209148

[ref68] Van Ooijen I. Fransen M. L. Verlegh P. W. Smit E. G. (2017). Packaging design as an implicit communicator: effects on product quality inferences in the presence of explicit quality cues. Food Qual. Prefer. 62, 71–79. doi: 10.1016/j.foodqual.2017.06.007

[ref69] Weisstein F. L. Choi P. Andersen P. (2019). The role of external reference price in pay-what-you-want pricing: an empirical investigation across product types. J. Retail. Consum. Serv. 50, 170–178. doi: 10.1016/j.jretconser.2019.05.017

[ref70] Woolley K. Kupor D. Liu P. J. (2023). Does company size shape product quality inferences? Larger companies make better high-tech products, but smaller companies make better low-tech products. J. Mark. Res. 60, 425–448. doi: 10.1177/00222437221124857

[ref71] Xia L. Monroe K. B. Cox J. L. (2004). The price is unfair! A conceptual framework of price fairness perceptions. J. Mark. 68, 1–15. doi: 10.1509/jmkg.68.4.1.427

[ref72] Zhao X. Lynch J. G. Chen Q. (2010). Reconsidering baron and Kenny: myths and truths about mediation analysis. J. Consum. Res. 37, 197–206. doi: 10.1086/651257

