# Supplementary material for: Psychological effects of horizontal price display: how left-right location shapes reference price and perceived quality
Source: Front Psychol. 2025 Feb 19;16:1497372. doi: 10.3389/fpsyg.2025.1497372 (PMC11879973; doi:10.3389/fpsyg.2025.1497372)
Supplement: Supplementary file 1 [file Data_Sheet_1.docx]

**Appendix A**


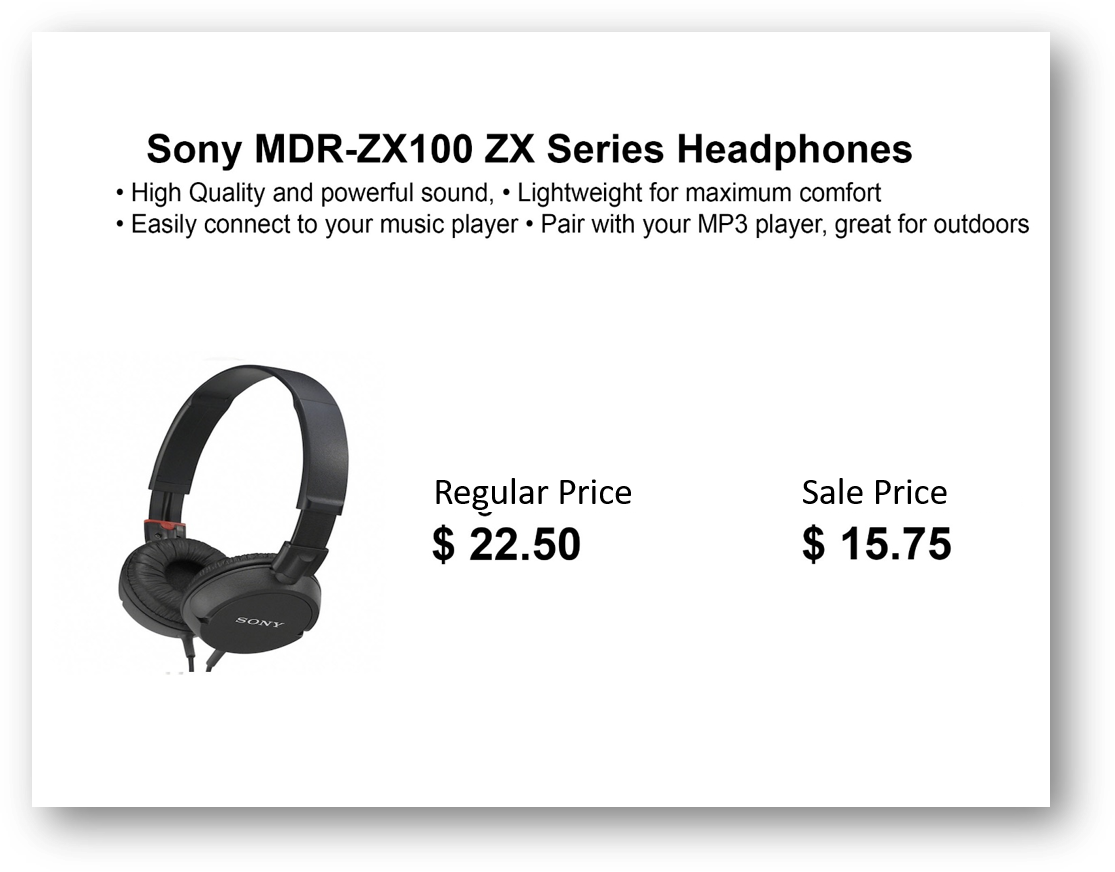
Stimulus used in Study 1A: Regular Price Left Condition

*Note: In the Discount Price Left Condition, the locations of the regular and* *discount prices were reversed.*

**Appendix B**

Stimulus used in Study 1b: Regular Price Left Condition.


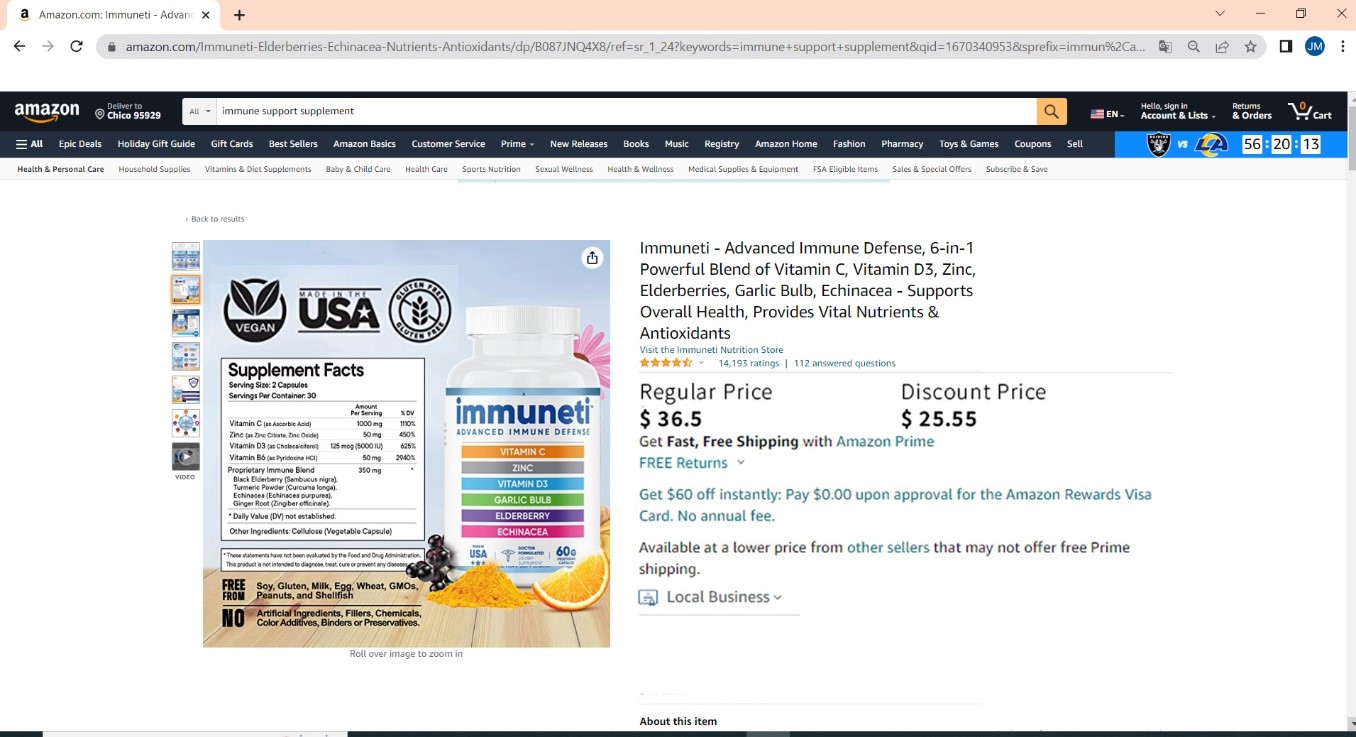
2A. With Quality Certification Marks

2B. Without Quality Certification Marks


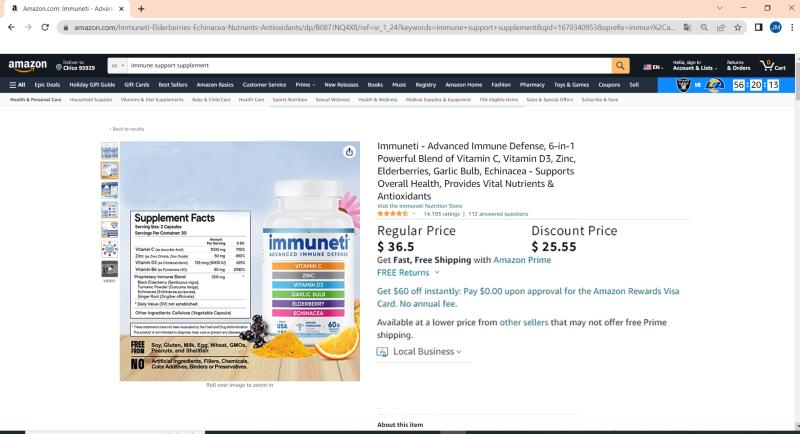


*Note: In the Discount Price Left Condition, the locations of the regular and discount prices were reversed.*

**Appendix C**


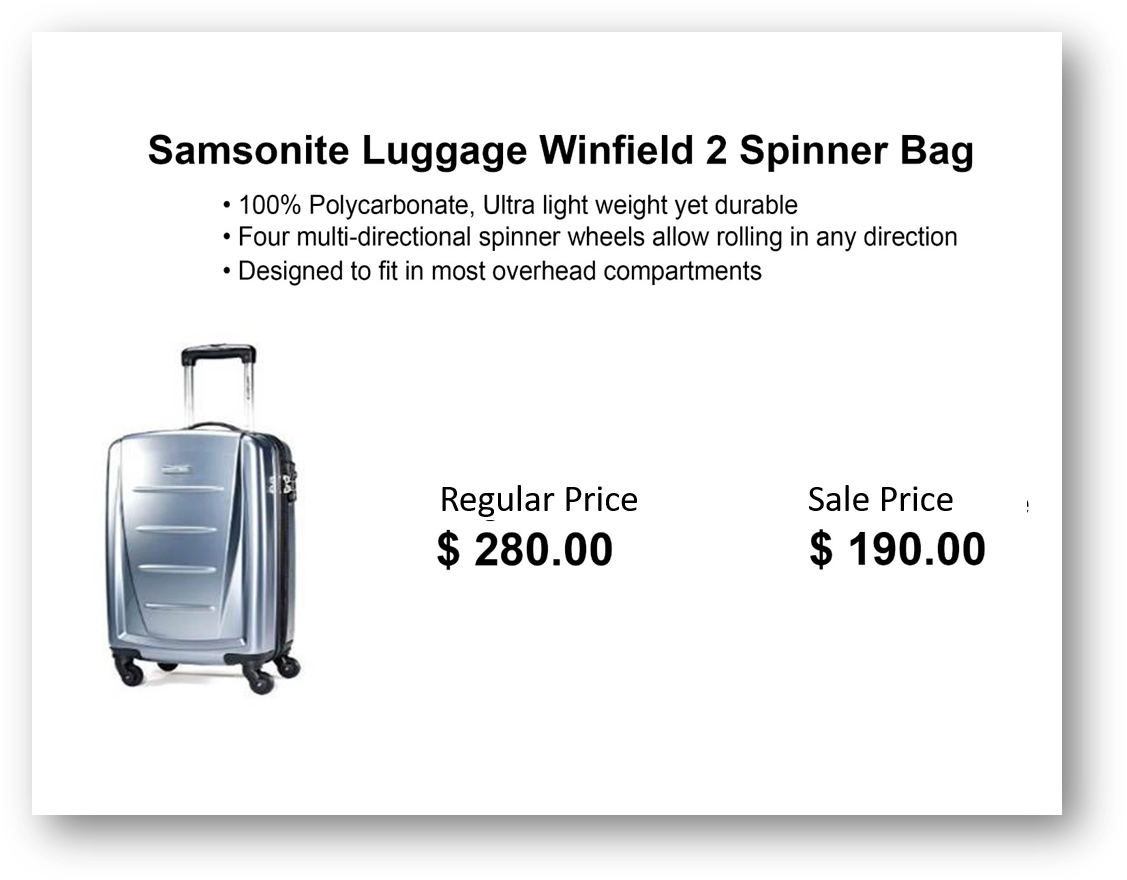
Stimulus used in Study 2: Regular Price Left Condition.

*Note: In the Discount Price Left Condition, the locations of the regular and discount prices were reversed.*
